# An Automatic Method for Generation of CFD-Based 3D Compartment Models: Towards Real-Time Mixing Simulations

**DOI:** 10.3390/bioengineering11020169

**Published:** 2024-02-09

**Authors:** Johan Le Nepvou De Carfort, Tiago Pinto, Ulrich Krühne

**Affiliations:** 1 Process and System Engineering Center, Department of Chemical and Biochemical Engineering, 2800 Kongens Lyngby, Denmark; jlne@kt.dtu.dk; 2 R/D Department, UNIBIO A/S, 4000 Roskilde, Denmark

**Keywords:** computational fluid dynamics, compartment model, digital twin, bioreactor

## Abstract

This article aims to develop a method to automatically generate CFD-based compartment models. This effort to simplify mixing models aims at capturing the interactions between material transport and chemical/biochemical conversions in large-scale reactors. The proposed method converts the CFD results into a system of mass balance equations for each defined component. The compartmentalization method is applied to two bioreactor geometries and was able to replicate tracer mixing profiles observed in CFD simulations. The generated compartment models were successfully coupled with, a simple Monod-type biokinetic model describing microbial growth, substrate consumption and product formation. The coupled model was used to simulate a four-hour fermentation in a 190 L reactor and a 10 $m^{3}$ reactor. Resolving the substrate gradients had a clear impact on the biokinetics, increasing with the scale of the reactor. Moreover, the coupled model could simulate the fermentation faster than real-time. Having a real-time-solvable model is essential for implementations in digital twins and other real-time applications using the models as predictive tools.

## 1. Introduction

Large-scale bioreactors are challenging systems to model due to the multiple phenomena taking place during fermentation. Bioreactor models should account for mass transport limitations (due to gas–liquid mixing and interfacial mass transfer) taking place at larger scales as well as chemical and biochemical conversions. Although biological growth typically occurs over a timescale of hours/days, biochemical reactions taking place inside the cell such as enzymatic reactions and allosteric regulation typically occur over a timescale of milliseconds/seconds [1,2,3]. Moreover, the consumption of substrates can occur on a timescale lower than the fluid circulation time in large-scale bioreactors, leading to the formation of spatial concentration gradients. To capture the interactions between material transport and chemical/biochemical conversions, models should include both phenomena. Computational fluid dynamics (CFD) offers a fine resolution of liquid/gas–liquid hydrodynamics in various fermentation equipment but is computationally expensive and requires long computational times to simulate short periods of equipment operating time [4,5]. This is due to the fine spatial discretization required to solve the mass and momentum equations. The high computational demand of CFD simulations is a clear limitation for applications where real-time model evaluations are required, e.g., in the case of model-based monitoring and control and implementation in digital twins. The computational demand and the difficult coupling with chemical reactions make it infeasible to simulate entire fermentations using CFD. The forecasting of large-scale fermentation processes can be used for model predictive control, where measurements of the process are used as inputs to the predictive model, and the predictions are used to actuate process accordingly to achieve optimal performance. For that purpose, simplified mixing models are required to include spatial heterogeneity to the real-time models.

An alternative approach to modelling material transport is through compartment modelling, a network of ideally mixed compartments with given volumes and exchange flowrates [6]. This approach is less computationally demanding, as the flow between compartments are given as an input to the model, and the mass and momentum balances are not included (avoiding the fine CFD discretization). The traditional approach to compartment modelling is to define the compartment network manually [6,7,8,9,10,11]. This step introduces a large amount of uncertainty to the model as the volumes and exchange flowrates are arbitrarily chosen to fit experimental data or based on a priori knowledge of the mixing behaviour of the system. More recent work aims at utilizing results from CFD simulations to formulate the compartment volumes and exchange flowrates [12]. Some articles use the CFD results to determine the exchange flowrates between manually set compartments [12,13,14,15,16,17], while others also use the CFD results to identify a 2D compartment network [18,19,20].

In this article, the proposed method aims at generating CFD-based three-dimensional compartment models applicable to any type of vessel geometry. The approach focuses on extracting the CFD results on a Cartesian grid, used as the basis of the compartment model. An additional step is added to the method, to enforce mass continuity of the generated compartment model. The developed method is then applied to two tank geometries, and the resulting compartment models are coupled with a Monod-type biokinetic model to simulate a full fermentation process and resolve potential concentration gradients originating from the top feeding of substrate.

## 2. Materials and Methods

### 2.1. Generating the Compartment Models

#### 2.1.1. Interpolation

The proposed method uses the results from a CFD simulation to automatically generate a uniformly gridded compartment model of the system with the resolution of the grid set by the user. To interpolate the CFD results on the defined grid, the CFD data are exported into a table containing cell centre data (x, y, and z positions; u, v, and w velocity components; eddy viscosity; and the volume of the element) with a row representing each CFD element. The geometric bounds are determined from the minimum and maximum values of the x, y, and z positions, and a Cartesian grid is defined within these bounds. The resolution of the grid in the three spatial dimensions is set by the user. For each grid cell, the velocity components are determined as the volume-weighted velocity average of the CFD elements present within the grid element. If the grid cell does not contain any CFD elements, the value is set to NaN to identify the specific geometry of the reactor. For the case where a grid cell inside the reactor does not contain any CFD elements (for the case of fine gridding relative to the CFD mesh) the velocity components are determined as the average value of the velocities in adjacent grid cells.

#### 2.1.2. Enforcing Continuity

**Figure 1:**
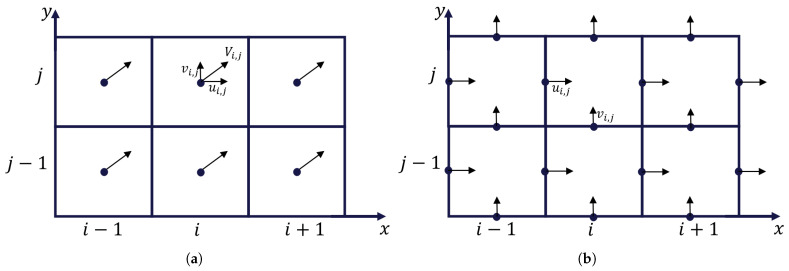
Schematics of two-dimensional collocated (**a**) and staggered (**b**) grids.

The first step is to convert the interpolated velocities in the grid into a staggered velocity grid, where the velocity between the grid elements is represented normal to each face (Figure 1). This representation is used to determine the velocities in and out of each grid cell, and will later be used to compute the flowrates between the grid cells. For the case of incompressible fluids, the continuity is ensured by forcing the divergence of the flow to zero at all points (Equation (1)) to avoid sink and source points in the velocity vector field [21].

(1) ∇·V=0

Here, the Gauss–Seidel method [22] is used to solve the continuity equation in a discretized space (Equation (2)) on the staggered velocity grid.

(2) $$\frac{\left(u_{i+1,j,k}-u_{i,j,k}\right)}{dx_{i}}+\frac{\left(v_{i,j+1,k}-v_{i,j,k}\right)}{dy_{j}}+\frac{\left(w_{i,j,k+1}-w_{i,j,k}\right)}{dz_{k}}=0$$

where $u_{i,j,k}$, $v_{i,j,k}$, and $w_{i,j,k}$ are the staggered velocities in the *x*, *y*, and *z* directions in grid cell [i,j,k] in $\left[\frac{m}{s}\right]$ and $dx_{i}$, $dy_{j}$, and $dz_{k}$ represent the dimensions of grid cell [i,j,k] in the *x*, *y*, and *z* directions in $\left[m\right]$.

The algorithm (Algorithm 1) loops through all grid cells changing the velocities around the grid cell by a factor of the divergence to solve for the continuity. If the divergence is positive, the velocities are forced in towards the cell, and if the divergence is negative, the velocities are forced outwards. This is repeated until the resulting divergence in the grid is lower than the tolerance level set by the user. In this work, all the results are generated with a tolerance for the root mean square of the divergence in all grid cells of $10^{-6}$. This usually requires a few thousand iterations for the cases presented here. A relaxation factor is used to facilitate the convergence of the algorithm, which was defined as 1.7 (strong over-relaxation). However, this value may be adjusted (between 0 and 2) by the user if the algorithm does not converge. The relaxation factor is the factor added to the change in variable values between iterations. A lower relaxation factor leads to a slower but a more stable convergence.

To identify the geometric bounds of the simulated tank, a variable *s* is defined in each grid cell, which takes a value of 0 if the velocity in the grid cell is not a number (NaN) and a value of 1 if the velocity is different from NaN. This will give a value of one to all grid cells inside the fluid domain and a value of 0 to all grid cells outside the fluid domain. The NaNs originate from the interpolation step, where if the grid cell does not contain any CFD element and is not filled, the velocity values in that grid cell will be NaN. The staggered velocities at the geometric boundaries of the tank are set to zero, ensuring no flow going in or out of the simulated fluid domain. The *s* variable is used when solving the continuity equation to account for cells and the boundaries of the tank and make sure the velocity at the tank walls remains zero.
**Algorithm 1** Solving the continuity equation in all grid cellsn←0**while** n>itr **do**    **for** i = 1 to $n_{x}$ **do**        **for** j = 1 to $n_{y}$ **do**           **for** k = 1 to $n_{z}$ **do**               **if** $s_{i,j,k}=1$ **then**                   $d\leftarrow \left(u_{i-1,j,k}-u_{i,j,k}\right)/\Delta x+\left(v_{i,j-1,k}-v_{i,j,k}\right)/\Delta y+\left(w_{i,j,k-1}-w_{i,j,k}\right)/\Delta z$                   $sn\leftarrow s_{i-1,j,k}+s_{i+1,j,k}+s_{i,j-1,k}+s_{i,j+1,k}+s_{i,j,k-1}+s_{i,j,k+1}$                   $u_{i,j,k}\leftarrow u_{i,j,k}+r\left(d/sn\right)s_{i-1,j,k}$                   $u_{i+1,j,k}\leftarrow u_{i+1,j,k}-r\left(d/sn\right)s_{i+1,j,k}$                   $v_{i,j,k}\leftarrow v_{i,j,k}+r\left(d/sn\right)s_{i,j-1,k}$                   $v_{i,j+1,k}\leftarrow v_{i,j+1,k}-r\left(d/sn\right)s_{i,j+1,k}$                   $w_{i,j,k}\leftarrow w_{i,j,k}+r\left(d/sn\right)s_{i,j,k-1}$                   $w_{i,j,k+1}\leftarrow w_{i,j,k+1}-r\left(d/sn\right)s_{i,j,k+1}$               **end if**           **end for**        **end for**    **end for**    n←n+1**end while**

#### 2.1.3. Converting Velocity Field to Volumes and Flows

The final step of the conversion from CFD to compartment model is to translate the continuity-corrected staggered velocities into flowrates by multiplying the velocity values with the area of the corresponding face (Equations (3)–(5)). Knowing that all grid cells are rectangular cuboids per definition, the area of the faces can be calculated as the product of the size of the cell in the two spatial dimensions of the face.

(3) $$Fx_{i,j,k}=u_{i,j,k}dy_{j}dz_{k}$$

(4) $$Fy_{i,j,k}=v_{i,j,k}dx_{i}dy_{j}$$

(5) $$Fz_{i,j,k}=w_{i,j,k}dx_{i}dy_{j}$$

In the same way, the volume of each grid cell can be calculated as the product of the size of the grid cells in the 3 spatial dimensions (Equation (6)).

(6) $$V_{i,j,k}=dx_{i}dy_{j}dz_{k}$$

where $V_{i,j,k}$ is the volume of the grid cell [i,j,k]. The entire conversion from the CFD table to the flow and volume matrices of the compartment model is implemented in Python as a single processing step requiring the following inputs:

- Grid resolution ($n_{x}\times n_{y}\times n_{z}$);
- Relaxation factor for the Gauss–Seidel method (default: 1.7);
- Tolerance level for the RMS of the divergence (default: $10^{-6}$).

### 2.2. Solving the System of Mass Balances

Knowing the volume of the grid cells, as well as the flowrates between adjacent cells, a system of material balances is formulated. For each grid cell, the direction of the flows is identified, in order to build the individual material balance equations. If the flow is leaving the grid cell, it is considered in the outgoing term of the balance equation, where it is accounting for material leaving the cell, whereas flows entering the grid cell are considered in the incoming term, accounting for the material entering the cell from adjacent cells. So, the amount of material leaving each grid cell due to convective transport ${\mathrm{OUT}}_{i,j,k}$ can be expressed as the of sum of outgoing flows multiplied by the material concentration in the cell and the amount of material entering each grid cell due to convective transport ${\mathrm{IN}}_{i,j,k}$ as the sum of the incoming flows multiplied by the material concentration in the corresponding adjacent cell (Equations (7) and (8)).

(7) $${\mathrm{IN}}_{i,j,k}=\sum_{a}^{A}F_{a}C_{a}$$

With *A* being all adjacent cells with flowrates entering the grid cell [i,j,k], $F_{a}$ being the flowrate entering the grid cell from the adjacent cell *a*, and $C_{a}$ being the material concentration in the adjacent cell *a*.

(8) $${\mathrm{OUT}}_{i,j,k}=\sum_{b}^{B}F_{b}C_{i,j,k}$$

With *B* being all adjacent cells with flowrates leaving the grid cell [i,j,k], $F_{b}$ the flowrate leaving the grid cell to the adjacent cell *b* and $C_{i,j,k}$ being the material concentration in the grid cell [i,j,k].

Due to the turbulent nature of the investigated system, eddy diffusivity is expected to have a significant impact on the total mass transport. Turbulent mass transport should therefore also be included in the compartment model. Mass diffusion in turbulent flow can generally be expressed as Equation (9) [21]. For the case of highly turbulent flows, the turbulent diffusion becomes significantly larger than the molecular diffusion, making the molecular diffusion negligible (Equation (10)).

(9) $$J_{i}=-\left(\rho D_{m,i}+\rho \frac{\mu_{t}}{Sc_{t}}\right)\nabla C_{i}$$

(10) $$J_{i}=-\left(\rho \frac{\mu_{t}}{Sc_{t}}\right)\nabla C_{i}$$

With $J_{i}$ being the diffusion flux of component *i*, ρ the density of the liquid, $D_{m,i}$ the kinematic diffusion coefficient of component *i* in the liquid, $\mu_{t}$ the eddy viscosity, $\nabla C_{i}$ the spatial concentration gradient of component *i* and $Sc_{t}$ the turbulent Schmidt number, defined as the ratio between eddy viscosity and eddy diffusivity. Experimental and numerical studies have shown that the turbulent Schmidt number can vary between 0.2 and 1.3 depending on the investigated system [23,24]. A value of 0.9 is chosen for this study, in agreement with the default value from the software ANSYS CFX used for the CFD simulations. This is to make the results between the CFD and generated CMs comparable; however, further tuning of the turbulent Schmidt number may be required to fit other systems.

The turbulent diffusion term is implemented in a discretized form (Equation (11) using the eddy viscosity values interpolated from the CFD and a specified turbulent Schmidt number.

(11) $$J_{i\to j}=-\left(\rho \frac{(\mu_{t,i}+\mu_{t,j})/2}{Sc_{t}}\right)\frac{C_{j}-C_{i}}{\Delta x}$$

With $J_{i\to j}$ being the diffusion flux of the transported component from cell *i* to cell *j*, $\mu_{t,i}$ and $\mu_{t,j}$ the eddy viscosity in cells *i* and *j*, $C_{i}$ and $C_{j}$ the concentration of the transported component in cells *i* and *j*, and Δx the distance between the two grid cell centres. The total turbulent diffusion from cell *i* to cell *j* can be determined by multiplying the resulting diffusion flux by the area between the two cells. Finally, the turbulent diffusion between all adjacent cells is added to the component mass balance (Equation (12)).

(12) $$TURB_{i,j,k}=\sum_{e}^{E}J_{e\to i,j,k}A_{e/i,j,k}$$

where $TURB_{i,j,k}$ is the net amount of transported material into the grid cell [i,j,k] due to turbulent diffusion, E represents all adjacent active grid cells, $J_{e\to i}$ is the diffusion flux of the transported compound from the adjacent grid cell *e* to the grid cell [i,j,k], and $A_{e/i,j,k}$ is the surface area between the adjacent grid cell *e* and the grid cell [i,j,k].

One last term *S* is included in the compartment model material balance, accounting for the sources of material in the grid cells. This term includes component reactions as well as source or sink points in the simulation. For example, a source point can be defined where material is added to the grid cell with a given rate. The total mass balance for a component *c* in cell [i,j,k] is shown in Equation (13).

(13) $$\frac{dC_{i,j,k}}{dt}=IN_{i,j,k}-OUT_{i,j,k}+TURB_{i,j,k}+S_{i,j,k}$$

The resulting system of material balance equations is implemented in Python and Julia to compare the performance of the two programming languages and to test their respective ODE solvers. For both programming languages, the script takes the generated compartment model object created in the previous step, and the mass balance equations are automatically constructed (using the above-described method, Section 2.2), and solved. The fastest solvers were chosen from the set of tested solvers; however, further work can focus on the performance of different numerical ODE solvers for this specific type of problem, to further reduce the computational time. The solvers used in this analysis are presented here: In Python, the system of equations is solved using the “solve_ivp()” function from the “Scipy” package with the “LSODA” solver. In Julia, the system of equations is solved using the “DifferentialEquations.jl” and “Sundials.jl” packages with the “CVODE_Adams()” solver. The two implementations in Python and Julia yielded systematically identical results. Therefore, only the results from the Julia simulations are presented in further results, unless stated otherwise.

### 2.3. Case Study: Stirred Tanks

The proposed method is applied on two reactor geometries (16 L and 190 L stirred tank reactors). The two studied tank geometries are summarized in Table 1. For each reactor geometry, a structured mesh is created using the ANSYS ICEM meshing tool with 521,344 elements for the 16 L reactor and 430,858 elements for the 190 L reactor (half geometry). The purpose of the case study is to compare the CFD simulations with the compartment model simulations. Therefore, no further validations of the CFD models are performed.

**Figure 2:**
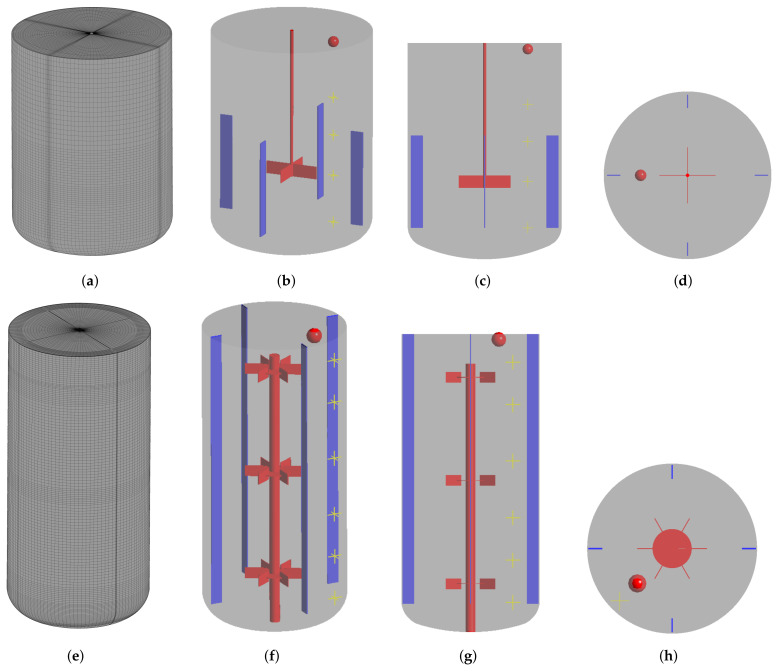
Geometry of the 16 L (top row) and 190 L (bottom row) reactors. The CFD meshes (**a**,**e**), iso views (**b**,**f**), view normal to the xy-plane (**c**,**g**) and view normal to the xz-plane (**d**,**h**) are shown. The baffles are shown in blue and the shaft/impeller(s) are shown in red. The red point indicates the position of the source point for the tracer injection and the yellow marks indicate the position of monitor points. Not to scale.

The CFD models of the two reactors are implemented in ANSYS CFX using the sliding mesh approach to model the impeller rotation. The fluid is modelled using the Eulerian approach as a continuous fluid with the properties of water and using the k−ϵ model for the turbulence modelling. The tank walls as well as the impeller and shaft surfaces are defined as no slip walls, and the top surface as a free slip wall to mimic the free surface of the fluid where the air drag is assumed negligible, and the surface shape assumed constant. Buoyancy is not accounted for in this study. The “High Resolution” advection scheme is applied together with a second-order backward Euler transient scheme. The regions surrounding the impellers are defined as rotating domains with a rotational velocity of 300 RPM. Transient simulations are performed for 40 s simulation time with a timestep of 0.001 s to ensure a fully developed flow and RMS Courant numbers below 10. To make sure the simulation had reached steady flow profiles, the impeller torque as well as the velocity values at multiple points in the tank are monitored during the simulation, and steady profiles were reached after 20 s for the 16 L reactor and after 5 s for the 190 L reactor. It should be noted that the 190 L tank show signs of steady oscillation due to the large scale turbulent nature of the studied system. Here, only the time-average velocity fields were used for further mixing studies; however, turbulent oscillations are known to contribute to a faster mixing, and should therefore be compensated for when choosing the turbulent Schmidt number. After the 40 s simulation time, the time-average velocity field of the last 15 s is used for the generation of the compartment model and to run a frozen flow field CFD simulation of mixing tracer injected as a pulse between 0 and 1 s at a source point in the top of the tank (Figure 2). The tracer is simulated as an additional variable governed by a species transport equation neglecting kinematic diffusion.

A similar simulation is performed with the generated compartment model with the same tracer injection pulse and rate and position in the top of the tank. The performance of the generated compartment models in reproducing material mixing can be assessed by comparing the resulting tracer concentration profiles from the CM and the CFD. The tracer concentrations are shown in dimensionless form, given by Equation (14) [25].

(14) $$C_{i}^{\prime }\left(t\right)=\frac{C_{i}\left(t\right)-C_{0}}{C_{\infty }-C_{0}}$$

where $C^{\prime }\left(t\right)$ is the dimensionless tracer concentration, C(t) is the tracer concentration, $C_{0}$ is the initial tracer concentration, and $C_{\infty }$ is the final/equilibrium tracer concentration. The degree of homogeneity in the tank can also be described as the variance $\sigma^{2}$ between the measured and final/equilibrium concentration for all monitor points *N* as described in Equation (15) [25].

(15) $$\sigma^{2}\left(t\right)=\frac{\sum_{i}^{N}{\left(C_{i}^{\prime }\left(t\right)-1\right)}^{2}}{N}$$

When simulating geometries with rotational periodicity or symmetries, the systems can be modelled as sections assuming theses symmetries. To showcase the ability of the presented method to generate a full compartment model from only a section of a CFD simulation, the 190 L tank is modelled as a half geometry, assuming rotational periodicity along the rotational axis. The CFD results are then mirrored along the symmetry axis to recreate the full geometry and generate a compartment model of the entire reactor. In this case, a secondary source point is added to the compartment model simulation to account for the symmetry of the tracer injection in the CFD simulation.

### 2.4. Coupling with a Biokinetic Model

To showcase the ability of the model to simulate the mixing in a reactor coupled with reactions, a simple Monod-type biokinetic model is implemented in the source term of the ODE system to simulate microbial growth in the tank, as well as substrate consumption and product formation. Such “blackbox” biokinetic models are often used to describe the microbial growth kinetics with only a handful of parameters and variables [26,27,28,29]. The ODE system is expanded to 3 components: biomass X, substrate S, and product P. The rate of formation of each component is described in Equations (16)–(19).

(16) $$\mu =\mu_{max}\left(\frac{S}{K_{S}+S}\right)$$

(17) $$r_{X}=\left(\mu -D_{X}\left(\frac{1}{S+1}\right)\right)X$$

(18) $$r_{S}=-\frac{\mu }{Y_{SX}}X$$

(19) $$r_{P}=\alpha \left(\frac{K_{P,1}}{2S^{3}+K_{P,1}}\right)\left(\frac{S}{K_{p,2}+S}\right)X$$

where $r_{X}$, $r_{S}$, and $r_{P}$ are the production rates of the biomass, the substrate, and the product, respectively; μ is the specific growth rate of the simulated microorganism; $\mu_{max}$ is the maximum specific growth rate; $K_{S}$ is the substrate limitation constant (or half-velocity constant); $D_{X}$ is the death rate of the microorganism; $Y_{sx}$ is the biomass to substrate yield; α is the product to biomass yield; $K_{p,1}$ is the substrate inhibition on product formation constant; and $K_{p,2}$ is the substrate limitation on product formation constant. The values of the biokinetic parameters are presented in Table 2. The biokinetic model is first solved on its own as a benchmark to compare with the results from the compartment model implementation. The biokinetic model alone is referred to as the 0D model. The same biokinetic parameters and initial values are implemented in the compartment model and solved for 4 h of bioreactor operating time. The impact of reactor scale on gradient formation is investigated by applying the compartment model to the 190 L tank and a scaled up version of the same reactor geometry of 10 $m^{3}$. The impeller rotational speed in the large tank is reduced to 100 RPM to maintain comparable impeller tip speeds in both reactors. For this case study, the reactors are operated as batch during the first hour of fermentation. After the first hour, substrate is added from the top. It is assumed that the substrate is added as a highly concentrated solution, and the working volume of the tank is constant. During the fed-batch operation, 0.05 $g\cdot m^{-3}\cdot s^{-1}$ substrate is added to the tank from the top.

## 3. Results

### 3.1. Case Study Mixing Time

**Figure 3:**
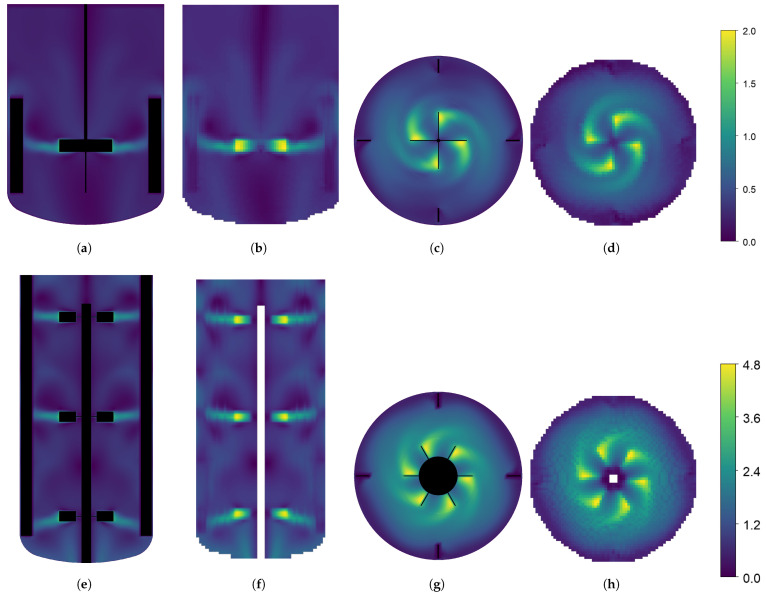
Velocity magnitude profiles on the xy-cut-plane through the middle of the tank, and on the xz-cut-plane through the middle of the impeller (middle impeller for the 190 L tank), reported in m/s. The results from the 16 L tank are shown on the top row and the results from the 190 L tank are shown in the bottom row. The CFD results are presented in subfigures (**a**,**c**,**e**,**g**) and the results from the generated (65×100×65) compartment model are presented in subfigures (**b**,**d**,**f**,**h**).

**Figure 4:**
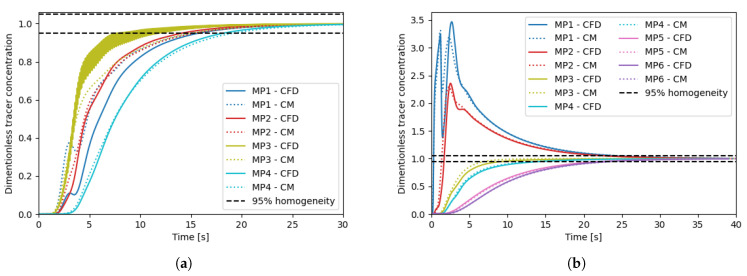
Tracer concentration profiles as function of time at different monitor points (shown in Figure 2) for the (**a**) 16 L reactor and (**b**) 190 L reactor. Solid lines show the results from the CFD simulations using frozen velocity field, and the dotted lines show the results from the generated compartment models of resolution (65×100×65). Dashed black lines show the 95% homogeneity.

The CFD simulations are performed in parallel on an Intel XeonGold6226R, 64 GB, 32 core computer, and the resulting time-averaged flow fields are used to generate the compartment models. All compartment model simulations are performed on a laptop computer (Intel i9, 32 GB) on a single core. This illustrated the simplicity of the generated compartment models relative to the CFD models, requiring much less computer power. The dimensionless simulation time of each simulation is reported in Table 3. Dimensionless simulation time is defined as the ratio between the wall clock time and the simulated time. A value above one will indicate the simulation is slower than real-time, and a value below one will indicate that the simulation is faster than real-time and can be used for forecasting. For each geometry, two compartment models are generated with grid resolutions of (35 × 60 × 35) and (65 × 100 × 65). A higher discretization on the y-axis is chosen to fit the overall geometry, and have similar spatial resolution in the three dimensions. Velocity magnitude profiles of the CFD results and the generated compartment models are shown in Figure 3. The tracer-mixing experiments were performed both with the CFD models, and the generated compartment models for the two reactor geometries and the concentration profiles at the different monitor points are reported in Figure 4. For the 16 L tank, no overshoot in the tracer concentration is observed at the four monitor points. The compartment model is able to reproduce similar concentration profiles, except for monitor point 1 (located in the top of the tank), which is overestimated by the compartment model.

**Figure 5:**
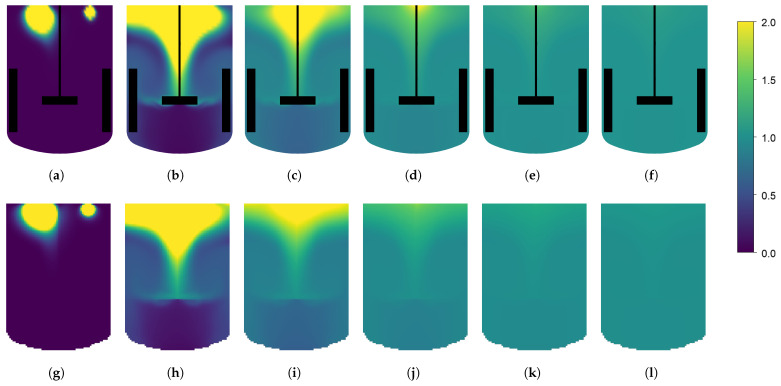
Dimensionless tracer concentration profiles in the xy-plane of the 16 L reactor. The top row shows the results from the CFD simulation and the bottom row show the results from the generated (65×100×65) compartment model. The results are shown after 1 s (**a**,**g**), 5 s (**b**,**h**), 10 s (**c**,**i**), 15 s (**d**,**j**), 20 s (**e**,**k**) and 25 s (**f**,**l**). Impeller and baffles in the CFD simulation are shown in black.

**Figure 6:**
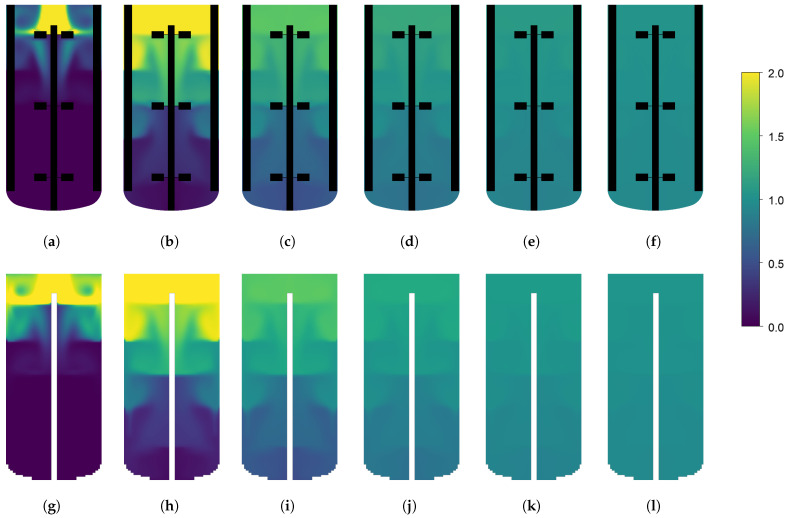
Dimensionless tracer concentration profiles in the xy-plane of the 190 L reactor. The top row shows the results from the CFD simulation and the bottom row show the results from the generated (65×100×65) compartment model. The results are shown after 1 s (**a**,**g**), 5 s (**b**,**h**), 10 s (**c**,**i**), 15 s (**d**,**j**), 20 s (**e**,**k**) and 25 s (**f**,**l**). Impellers and baffles in the CFD simulation are shown in black.

**Figure 7:**
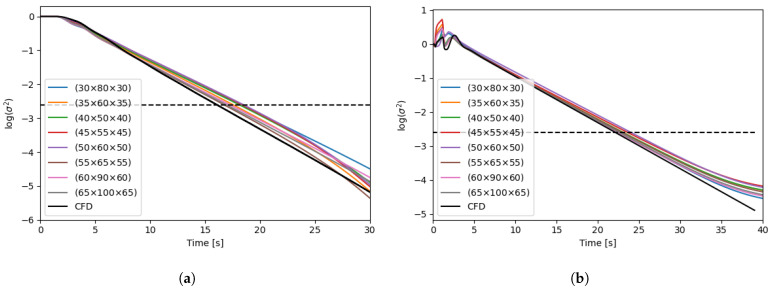
Degree of homogeneity $\log(\sigma^{2})$ as function of time for the generated compartment models with various resolutions (see labels) for the 16 L tank (**a**) and the 190 L tank (**b**). Results from CFD are shown in black. The dashed line shows the 95% homogeneity.

This could be explained by the numerical error related to using a larger element size for the simulation. Also, the CFD results for monitor point 3 show a strong oscillation, which is not present in the results from the compartment model. As both the CFD and CM simulations were performed with frozen flow fields, the oscillations are not an artefact of the turbulent fluctuations in the flow. The oscillations could rather be explained by a finer resolution of small vertices in the CFD. Overall, both the CFD and the CM predict a mixing time around 18 s. For the 190 L tank, a clear overshoot of the tracer concentration is observed in the top of the tank. This is expected in a larger stirred tank with multiple impellers, where the axial mixing can be quite poor. Here, the CM is in good agreement with the CFD model and is able to reproduce similar tracer concentration profiles. The overall mixing time is predicted around 25 s. The tracer concentration profiles on the xy-plane at different time points are shown in Figure 5 and Figure 6. Similar tracer concentration profiles are observed between the results from the CFD and CM simulations. It can thus be concluded that the compartment model is able to predict mixing times accurately as well as the dynamic concentration profiles during mixing. Further discretization study can help to determine the optimal grid resolution giving the fastest simulation with satisfactory results. A set of compartment models with various resolutions are generated for each tank geometry, and for each compartment model, the tracer mixing simulation is performed. The resulting mixing profiles are shown in Figure 7 as the degree of homogeneity $\log(\sigma^{2})$. The CFD simulations systematically yielded lower mixing times and the coarser the grid, the slower the mixing. Interestingly, this goes against the idea that coarse discretization leads to higher numerical diffusion and therefore faster mixing. Overall, the compartment models were all able to predict similar mixing times (±4 s). It can also be noted that the homogeneity profiles in the first 5 s of simulation are quite different for the different grid resolutions, for the case of the 190 L tank. Here, low resolution grids are not able to accurately resolve the first few seconds of the mixing while providing satisfactory homogeneity profiles only after the first 5 s. In the presented results, the velocity field is considered frozen both for the CFD and CM simulations. The time-average flow field was selected for further study, neglecting the turbulent time dependent fluctuations in the flow. Transient fluctuations in the flow are known to have an impact on the mixing of dilute species, typically reducing the mixing time. Further work should focus on including the time dependent fluctuations of the flow field into the generated compartment models to better reproduce the transient mixing behaviour of the system.

The generated compartment models were able to reproduce numerical mixing simulations in good agreement with the results from the pure CFD simulations. The fine resolution of CFD meshes are important to resolve flow phenomena such as velocity gradients and turbulence. However, reducing the resolution of the discretization does not seem to have a significant impact on the overall mixing profiles, thus making these coarser models applicable for mixing simulations that do not require to solve the fluid dynamics.

Moreover, the compartment models greatly reduced the computational complexity of the simulation, allowing for faster model evaluations, in some cases faster than real-time (CM Julia (35×60×35), Table 3). This makes the models applicable in solving computationally expensive problems, such as optimisations and sensitivity analysis, and integration into multi-scale models for plant-wide and production line modelling. The fast model evaluations could also allow the model to be used in real-time applications such as digital twins. The fast model evaluations, as well as the ability to use customable stiff numerical solvers makes it possible to solve for multiple species and implement other models such as chemical or biochemical reaction models. This is investigated further in the following section.

### 3.2. Simulation of a Biological Process

**Figure 8:**
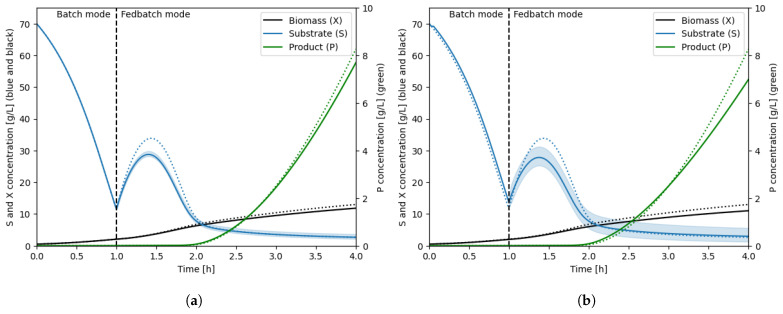
Concentration profiles of biomass, substrate, and product in the bioreactor for the 190 L tank (**a**) and 10 $m^{3}$ tank (**b**). Results from the 0D simulation are shown as dotted lines, the mean concentration profile from the compartment model simulation in solid lines, 95% of the concentrations are within the coloured area.

A (30 × 40 × 30) grid compartment model is generated for the 190 L and 10 $m^{3}$ tanks based on the CFD results. As described in Section 2.4, the system of ODEs is expanded to three components, and the biokinetic model (Equations (16)–(19)) is implemented in the respective source terms of the material balances, in all grid elements. The coupled system is used to simulate a 4 h fermentation, and the resulting concentration profiles are presented in Figure 8. Here, 4 h of simulation time was enough to see the overall dynamic of the process and assess the performance of the compartment models; however, real fermentations typically take a longer time.

During the batch operation, where substrate is not supplied, the system is expected to remain homogeneous and follow the 0D fully homogeneous model. When switching to fed-batch mode, where substrate is supplied from the top of the tank, the compartment models begin to exhibit substrate gradients and the average concentration profiles start deviating from the 0D model. The 10 $m^{3}$ shows a larger deviation from the 0D model compared to the 190 L tank, which is expected due to the larger mixing time in the large tank. A wider distribution of substrate concentrations can also be observed for the 10 $m^{3}$ tank. This is observed from the width of the blue region around the substrate concentration curves (Figure 8) representing the range of concentrations observed in the tank. The greater substrate gradients observed in the large tank explain the slower growth and slower product formation, due to the kinetic behaviour of the microorganism (inhibition and limitation terms in Equations (16) and (19)). The 10 $m^{3}$ reactor shows a 12% drop in product formation after four hours relative to the 0D model. This value is specific to the implemented biokinetic model, and it should be noted that the magnitude of the impact of gradients highly depends on the investigated microorganism and its sensitivity to changing surroundings [1].

## 4. Discussion

The results presented in this article showcased the ability of the developed compartment models to perform dynamic mixing simulations based on CFD results and replicate the mixing behaviour. Only solving the mass balance equations, the compartment models are less computationally demanding compared to the CFD models and can be solved much faster (2200 times faster than CFD with the (35×60×35) grid compartment model solved in Julia). This allows for coupling with more complex reacting systems, with more components and stiff reaction kinetics. In Section 3.2, a three-component biological system coupled to the mixing model was used to simulate 4 h of fermentation in 2.08 h wall clock time. Having the ability to simulate entire fermentation processes, the models can be used to predict gradients and how the mixing will affect the entire process. This is useful for exploring and optimizing tank design and operation to minimize gradients and extreme exposures near inlets. For instance, one can investigate the pH surrounding a base inlet or substrate gradients based on the feeding and monitoring strategies. As observed in the results Section 3.2, larger tanks are prone to slower mixing times, and will exhibit larger gradients. It is therefore important to consider the impact of mixing on the process when designing large scale equipment. Moreover, the compartment models can be used to identify zones in the reactors operating under different conditions, and use the results to design scale-down experiments of the large tank and replicate the large scale gradients in a lab-scale setup. The developed compartment models can also be used to predict the behaviour of a fermentation based on measurements of the system and the real-time solutions provided by the models. For example, the models can be used to early diagnose failed batches, or for model-based monitoring and control of the process.

It should be noted that the generated compartment models are based on a single time-averaged CFD solution. The compartment models will therefore have a fixed volume and fixed flowrates between compartments. On that note, the hereby presented method using a frozen flow field would be more accurate for systems with low fluctuations in the flow field, and the error between the presented method and the physical is expected to increase for systems with larger fluctuations in the flow. Large flow oscillations typically occur in the transition flow regime between laminar and fully turbulent flows. Further implementation of transient fluctuations in the flow fields will therefore provide more realistic results, especially in large-scale reactors where oscillating flow patterns may occur. With regards to the constant volume, the compartment models can only be used to simulate batch, quasi-batch, and continuous operations. Quasi-batch refers to a fed system with assumed constant volume, for example a batch fermentation with base addition for pH control, or aeration. Simulating a fed batch with changing volume may be achieved by discretizing the volume change and provide multiple CFD results with different filling levels. A similar approach was performed in 2D in a previous work [30].

Due to the fixed flow in the compartment models, changes in the flow conditions (e.g., agitation, viscosity) will require new CFD input. A workaround is to produce a range of CFD results at different conditions and create reduced-order models to describe the flow in the compartments as a function of the flow input parameters [31]. Further work may also focus on including the energy balance in each compartment to solve for the temperature.

Further expanding the automatic compartment model generation to account for multiphase systems will allow for simulating mass transfer in aerated fermentations and couple interfacial mass transfer limitations to the mixing and biokinetic models. Coupling with more advanced biological models may also be considered, for instance genome-scale models, to investigate the impact of the gradients on the metabolic regime of the microorganisms.

## 5. Conclusions

In this article, an automatic method for generating CFD-based compartment models is proposed and tested. Compartment models are generated for two tank geometries and used to perform dynamic mixing simulations. For both geometries, the compartment models yielded mixing times and profiles similar to the CFD simulations in a fraction of the time needed for a CFD simulation. The input parameters for the automatic method are discussed, and it is concluded that the grid resolution can have a significant impact on the results and should be selected with care. A 4 h fermentation was successfully simulated by coupling the generated compartment models with a simple biokinetic model, and the model was able to resolve concentration gradients and their impact on the overall process. This approach may also be used outside the scope of simulating bioprocesses to all stiff systems, where coupling of transport phenomena and reactions is required (e.g., chemical reactors, separation units).

The method was designed to be as generic as possible, and it can handle CFD results from any software with any type of geometry and mesh. The generated models can resolve heterogeneities and predict dynamic responses to changes in the process operating conditions in real time, making the generated models useful for a wide range of applications, such as design optimization, monitoring, and control as well as applications in digital twins.

## Figures and Tables

**Table 1:** Geometric properties of the 16 L and 190 L reactors.

Parameter	Reactor #1	Reactor #2
Total Volume	16.355 L	189.947 L
Inside height	35 cm	105.5 cm
Bottom type	rounded	rounded
Inside diameter	25 cm	48.8 cm
Impeller diameter	8.5 cm	20 cm
Number of impellers	1	3
Impeller type	Paddle	Rushton
Number of blades on impeller	4	6
Distance from top to impeller centre	22.5 cm	15.375, 51.875, 88.375 cm
Number of baffles	4	4
Baffle width	2 cm	4 cm
Baffle height	15 cm	95.5 cm
Agitator shaft diameter	5 mm	3.5 cm

**Table 2:** Biokinetic parameters and initial conditions.

Parameter	Value	Unit
$\mu_{max}$	2	$h^{-1}$
$K_{S}$	10	$g\cdot L^{-1}$
$D_{X}$	5 × $10^{-5}$	$g\cdot m^{-3}$
$Y_{sx}$	0.03	$\mathrm{gX}\cdot {\mathrm{gS}}^{-1}$
α	2.8 × $10^{-4}$	$\mathrm{gP}\cdot {\mathrm{gX}}^{-1}$
$K_{P,1}$	100	$g\cdot m^{-3}$
$K_{P,2}$	1	$g\cdot m^{-3}$
Initial condition	Value	Unit
X0	0.5	$g\cdot L^{-1}$
S0	70	$g\cdot L^{-1}$
P0	0	$g\cdot L^{-1}$

**Table 3:** Dimensionless simulation time (defined as wall clock time/simulated time) of the CFD and CM tracer mixing simulations.

Reactor	ANSYS-CFX 2021R1 CFD Mesh	CM Python (35×60×35)	CM Julia (65×100×65)	CM Julia (35×60×35)
16 L	1455	22.59	10.05	0.641
190 L	682	4.927	10.22	0.722

## Data Availability

Data are contained within the article.

## Abbreviations

The following abbreviations are used in this manuscript:

CFD	Computational Fluid Dynamics
CM	Compartment Model
NaN	Not a Number
RMS	Root Mean Square
ODE	Ordinary Differential Equation
RPM	Rotations Per Minute
0D	Zero Dimensional
2D	Two Dimensional
3D	Three Dimensional
